# Harnessing downstream NF-*κ*B signalling to achieve apoptosis-inducing anti-cancer-specific activity

**DOI:** 10.1038/cddis.2016.221

**Published:** 2016-07-21

**Authors:** M Vaccarezza, M Vitale

**Affiliations:** 1 School of Biomedical Sciences, Faculty of Health Sciences, Curtin University, Kent Street, Bentley, Perth, WA 6102, Australia; 2 Department of Biomedical, Biotechnological and Translational Sciences (S.Bi.Bi.T.), University of Parma School of Medicine; CoreLab Facility, University Hospital of Parma, Via Gramsci 14, Parma 43126, Italy

Transcription factors of the NF-*κ*B family are driven by many inflammatory stimuli and activate expression and production of soluble cytokines, chemokines and inflammatory mediators, and a plethora of important immune response genes.^1^

It has been known since long time that NF-*κ*B is frequently activated in solid and blood cancer,^2, 3^ stemming the idea that its oncogenic function relays on inhibition of cancerous cell death, driven either by antitumor drugs or tumour-suppressor mechanism.^3^ This hypothesis, robustly based on experimental models, originated the valid assumption that inhibiting NF-*κ*B activity should be an effective therapeutic tool alone or in combination with chemotherapy or radiation therapy. This hypothesis ignited an intense race to develop NF-*κ*B inhibitors, most often by targeting I-*κ*B kinase, a protein kinase that is essential for NF-*κ*B activation.^4^ When such experimental inhibitors were tested in animal experimental settings or in human volunteers, they elicit several unwanted side effects, such as inflammation, immune disfunction and liver tissue damage. These side effects were in part anticipated by knock-out gene studies in mice, whereas the inflammatory effect was rather unexpected. The mechanism behind this action was linked to the NF-*κ*B inhibitory activity of Caspase 1 activation, the latter being an essential step in secretion of IL-1-*β* by activated macrophages.^4^ The clinical development of IKK/NF-*κ*B inhibitors was thus halted.

Recent studies by Tornatore *et al.*^5^ have intriguingly re-opened the possibility to achieve NF-*κ*B specificity modulation in oncology, tackling a novel approach to reach this important goal. The studies by Tornatore *et al.*^5^ stem by the fact that NF-*κ*B mediates its survival and anti-apoptotic function by preventing the activation of the pro-apoptotic protein kinase JNK. *In vitro* experiments showed that this inhibitory action on JNK activation is mediated by the stress-induced protein GADD45*β*.^6, 7^ The latter protein interacts with the JNK activating kinase MKK7 inhibiting its catalytic activity.^8, 9^ Previous results from GADD45*β* knock-out mice revealed that targeting this protein has no effect on overall health, pointing to a redundant role on normal cell and, at the same time, a potential feasible target on cancerous cells.^10^ Tornatore *et al.*^5^ probed GADD45*β* expression in various cancers and found it to be highly elevated in several tumours, and especially in multiple myeloma (MM) samples. MM is known to have several mutations that lead to constitutive and continuous NF-*κ*B activation. Primary MM samples (as well as several MM cell lines) demonstrated that GADD45*β* expression is relevant in 50% of clinical cases and silencing of the gene in cell lines resulted in substantial cell death. Of note, apoptosis depended on MKK7-driven JNK activation, underscoring the potential of a molecule that could interfere with the capacity of GADD45*β* to downregulate MKK7 activity. Such a molecule would work as a specific inhibitor of GADD45*β*-induced cell survival. Tornatore *et al.*^5^ took a herculean effort in order to design inhibitors of GADD45*β*-MKK7 interaction and found two tetra-peptides that showed activity at low concentration. Interestingly even the D-enantiomers of both peptides were substantially active, thus bypassing animal and human serum protease potential harmful degradation and deactivation. Subsequent molecular design refinement originated DTP3, that is a very high affinity and selective MKK7 binder, driving to a sustained JNK activation and consequent death of MM cell lines expressing GADD45*β* (as outlined in Figure 1). Tornatore *et al.*^5^ also demonstrate strong anti-tumour activity in primary cells obtained from MM patients with very low non-specific toxic activity, rendering the new compound very appealing and potentially with a remarkable high therapeutic index.^5^ *In vivo* experiments convincingly confirmed the *in vitro* findings, with no sign of systemic toxicity for the new compound and potential synergic activity with other drugs already utilised in clinical setting.^5^ Further studies lead to the successful development of the compound that is now ready for clinical trials.^11^ Of note, pharmacodynamics assessment suggests a clinical dose three times/week i.v., testing range doses from 0.5 to 20 mg/kg. These data support also a potential subcutaneous route of administration and also a 2 days/week bolus regimen.^11^ Taken together, the preclinical studies show outstanding pharmacology properties and robust selectivity parameters. Wisely, the developers started a companion specific biomarker programme (based on the GADD45*β* expression), in order to optimise clinical development and delivery appropriateness.^11^ More clinical studies are needed to finally validate this novel modulation pathway, to verify long-term activity of DTP3, potential drug-resistance pathways and combination therapy possibilities, but nevertheless the achievement of Tornatore *et al.*^5^ is of paramount value. Not only we can finally have a much more specific way to harness NF-*κ*B signalling, but we could envision this apoptosis-inducing mechanism in other haematologic and solid tumours. Even in presence of redundant NF-*κ*B pathways that likely sustain anti-apoptotic activity in cancer cells, DTP3 could be used in combination therapy with several compounds targeting known and new signalling cascades. Previous experiments using different lead molecules combined with established drugs corroborate this hypothesis.^12^

Previous efforts to target the NF-*κ*B complex have failed to preserve the wide functions of NF-*κ*B signalling, and have thus been limited by serious toxicity. By exploiting the GADD45*β*/MKK7 complex it would be realistic to inhibit the cell survival axis of the NF-*κ*B signalling complex in cancer cells to achieve clinical activity with a broad therapeutic window.

## Figures and Tables

**Figure 1:**
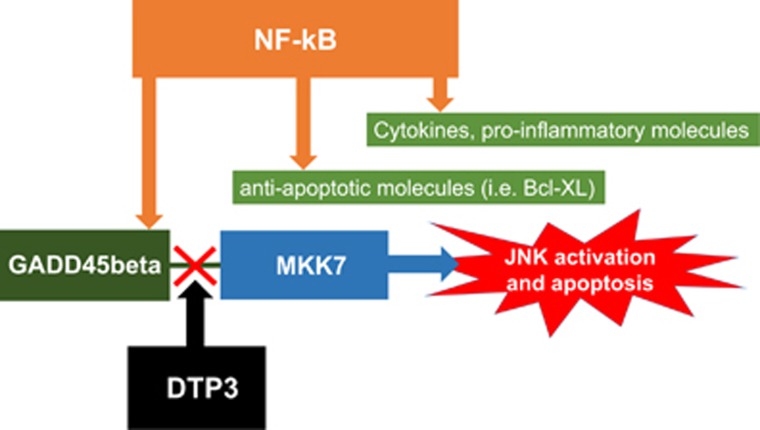
NF-*κ*B downstream signalling and DTP3 action
